# Dataset on woody aboveground biomass, disturbance losses, and wood density from an African savanna ecosystem

**DOI:** 10.1016/j.dib.2022.108155

**Published:** 2022-04-11

**Authors:** Liana Kindermann, Magnus Dobler, Daniela Niedeggen, Ezequiel Chimbioputo Fabiano, Anja Linstädter

**Affiliations:** a Biodiversity Research / Systematic Botany, Institute of Biochemistry and Biology, Faculty of Science, University of Potsdam, Maulbeerallee 1, Potsdam 14469, Germany; b Grassland Ecology and Grassland Management, Institute of Crop Science and Resource Conservation (INRES), Agricultural Faculty, University of Bonn, Bonn 53115, Germany; c Terrestrial Ecology Group, Institute of Zoology, Department of Biology, Faculty of Science and Mathematics, University of Cologne, 50674 Cologne, Germany; d Department of Wildlife Management and Tourism Studies, University of Namibia Katima-Mulilo Campus, Ngweze, Katima-Mulilo 1096, Namibia

**Keywords:** Damage assessment, Disturbance impacts, Disturbance indicator, Elephant disturbance, Tree allometry, Specific wood density, Woody aboveground biomass, Wood specific gravity

## Abstract

This dataset comprises tree inventories and damage assessments performed in Namibia's semi-arid Zambezi Region. Data were sampled in savannas and savanna woodlands along steep gradients of elephant population densities to capture the effects of those (and other) disturbances on individual-level and stand-level aboveground woody biomass (AGB). The dataset contains raw data on dendrometric measures and processed data on specific wood density (SWD), woody aboveground biomass, and biomass losses through disturbance impacts. Allometric proxies (height, canopy diameters, and in adult trees also stem circumferences) were recorded for *n* = 6,179 tree and shrub individuals. Wood samples were taken for each encountered species to measure specific wood density.

These measurements have been used to estimate woody aboveground biomass via established allometric models, advanced through our improved methodologies and workflows that accounted for tree and shrub architecture shaped by disturbance impacts. To this end, we performed a detailed damage assessment on each woody individual in the field. In addition to estimations of standing biomass, our new method also delivered data on biomass losses to different disturbance agents (elephants, fire, and others) on the level of plant individuals and stands.

The data presented here have been used within a study published with Ecological Indicators (Kindermann et al., 2022) to evaluate the benefits of our improved methodology in comparison to a standard reference method of aboveground biomass estimations. Additionally, it has been employed in a study on carbon storage and sequestration in vegetation and soils (Sandhage-Hofmann et al., 2021).

The raw data of dendrometric measurements can be subjected to other available allometric models for biomass estimation. The processed data can be used to analyze disturbance impacts on woody aboveground biomass, or for regional carbon storage estimates. The data on species-specific wood density can be used for application to other dendrometric datasets to (re-) estimate biomass through allometric models requiring wood density. It can further be used for plant functional trait analyses.

## Specifications Table

**Table:** 

Subject	Biology; Plant Science: General
Specific subject area	Savanna ecology; disturbance ecology; global change ecology; disturbances impacting woody aboveground biomass
Type of data	Table Image Graph Figure
How the data were acquired	We stratified our sampling into two vegetation types (savanna and savanna woodland) and three levels of elephant population densities (high, medium and low); for details see [2]. Each stratification level was sampled with ten replicate plots per site (*n* = 60). A systematic but flexibly attunable plot design was employed; for details see [1]. Basic tree size proxies (height, stem circumferences, canopy diameters) were taken in the field with the aid of meter sticks and measuring tapes. For very large trees, a clinometer PM-5 by Suunto was used for height determination. A two-threaded increment borer by Haglöf Sweden (inner diameter 5.15 mm) was used for extraction of wood cores from tree stems while in shrub species, a saw was used to cut stem pieces [3]. Fresh wood samples were measured in length by using a meter stick and a total of five diameter readings on each wood sample were taken with calipers. Wood samples were first air-dried and later dried in standard laboratory drying ovens at 105°C until no further weight reduction was detected [3]. Their weight was determined using standard laboratory fine scales (with a division of 0.001g).
	Field measurements were digitalized in MS Excel, and data preparation including all estimation procedures for aboveground biomass were conducted in a spreadsheet. Data analysis was conducted with opensource software R [4] and RStudio [5], using packages dplyr [6], rstatix [7], and reshape2 [8]. Data exploration was performed visually using R packages ggplot2 [9], and scales [10].
Data format	Raw Analyzed Processed
Parameters for data collection	Data collection considered all woody tree and shrub species and individuals of all sizes, age classes, and damage levels were recorded with dendrometric proxies (height and canopy diameter). For adult-sized stems (> 15 cm basal circumference), additional stem circumference readings were taken, see [1] for details. Specific wood density was measured in 2-10 healthy, mature individuals per species, depending on species’ abundance.
Description of data collection	On all plots, woody individuals of all species, sizes, and damage levels were measured non-destructively. For each sampled individual, we recorded (i) species identity, (ii) height, and (iii) canopy diameters. On living stems > 15 cm circumference at the base we recorded (a) basal circumference, (b) circumference at 130 cm aboveground, and where (a) or (b) was impossible (c) a stem circumference at an alternative section of the stem and its corresponding height. Dead stems were measured at the base if living regrowth was present. A representative number of individuals in each species was sampled for wood density measurements.
Data source location	Institution: University of Potsdam – Institute of Biochemistry and Biology, Faculty of Science, University of Potsdam, Germany; Collaborative Research Center TRR228 ‘Future Rural Africa’, project A01 (‘Future Carbon Storage’) Region: Zambezi Region; National Parks Mudumu and Bwabwata as well as Communal Conservancies Wuparo and Mashi Country: Namibia Latitude and Longitude: 18°04.000′S; 23°25.000′E (see detailed coordinates for each plot in data file)
Data accessibility	Repository name: Mendeley Data Data identification number: DOI: 10.17632/3cs85wd3gb.5[11] Direct URL to data: http://dx.doi.org/10.17632/3cs85wd3gb.5
Related research article	L. Kindermann, M. Dobler, D. Niedeggen, A. Linstädter, A new protocol for estimation of woody aboveground biomass in disturbance-prone ecosystems. Ecol. Indic. 135 (2022) 108466. https://doi.org/10.1016/j.ecolind.2021.108466

## Value of the Data

• The data provide dendrometric measurements and estimates of woody aboveground biomass (AGB) as well as AGB losses from savanna and savanna woodland sites in north-eastern Namibia that differ in elephant population densities, and also in the level of damages by other disturbance agents including fire. The data are important to assess tree and shrub biomass in disturbance-prone ecosystems and disturbance impacts on woody vegetation.
• Data are useful for dryland ecologists, global change ecologists, or conservation biologists interested in the effects of elephant and fire disturbances on woody vegetation. They can also be of interest for carbon storage assessments.
• Data can be exploited to analyze structural and compositional characteristics of the study area, providing e.g., information for national or regional conservation policies.
• Data can also provide useful information to compare the pros and cons of the adoption of a new method [1] to record AGB of highly disturbed woody plants in disturbance-prone ecosystems.
• Data on tree species’ ‘specific wood density’ (SWD) may be used to (re-) analyze existing dendrometric datasets from the region with new allometric equations requiring this proxy e.g. [12].

## 1 Data Description

*Data Table***:** The dataset presents the results of tree inventories conducted along steep gradients of elephant disturbances in African savannas and woodlands located in Namibia's semi-arid Zambezi Region (18°04.000’S; 23°25.000’E). Data were collected in 60 plots (25 × 40 m). The data table contains six spreadsheets: a basic information spreadsheet, a detailed legend, and four data spreadsheets. The first data spreadsheet (data prop) contains aboveground biomass data as derived with our proposed method for n = 6,179 trees and shrubs on 60 plots, distributed over four sites, two vegetation types, and three levels of elephant density. Several aboveground biomass (AGB) partitions are given for each woody individual: the individually estimated AGB, the individual's AGB scaled to a unit per area basis [kg ha^−1^], and AGB losses to various recorded disturbance agents (elephants, fire, woodcutting, browsers other than elephant, abiotic stress). The second data spreadsheet contains mean specific wood density (SWD; also known as ‘wood specific gravity’ [3]) values per species as derived from our measurements. The third data spreadsheet contains the raw data of dendrometric proxies taken in n = 6,179 trees from which AGB values in ‘data prop’ have been calculated, see [1] for details. The last spreadsheet contains geographical coordinates for each of the *n* = 60 plots.

**Fig. 1:**
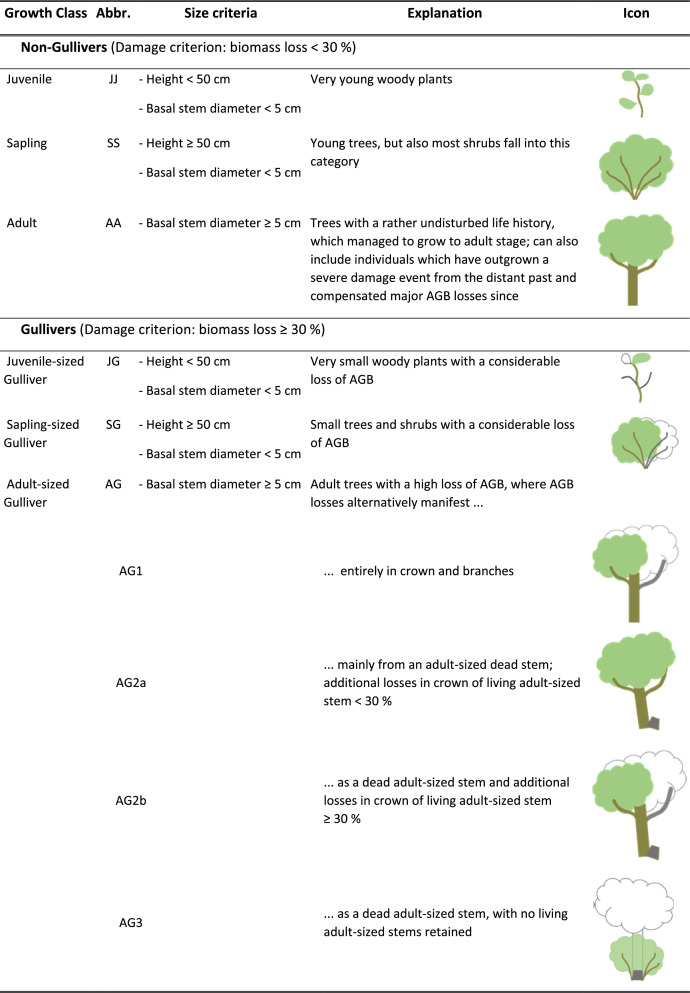
The six growth classes with sub-types and the metric criteria they are defined by.

Fig. 1: In Fig. 1 we describe and illustrate the six growth classes with sub-types and list the metric criteria they are defined by.

**Fig. 2:**
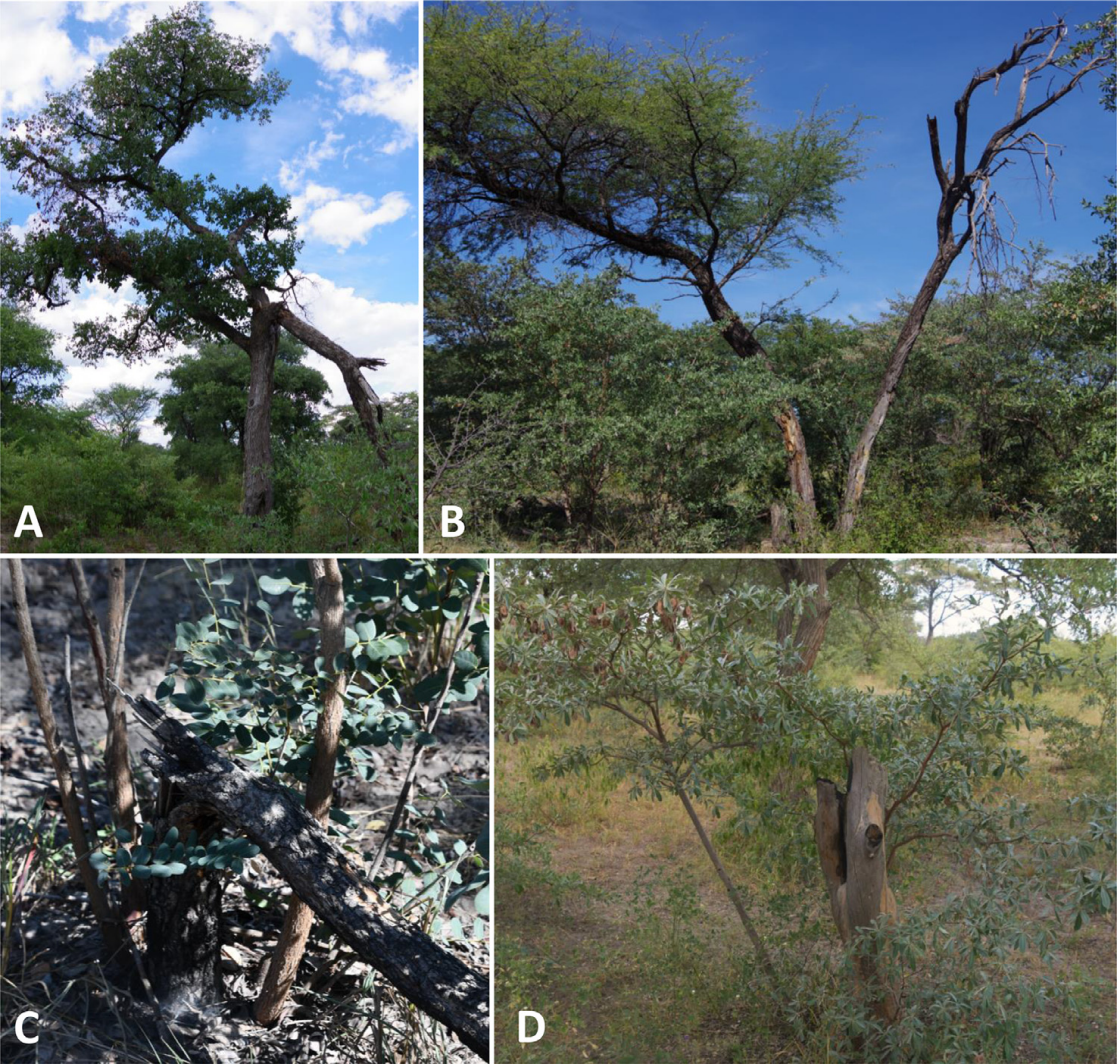
Photographic examples for the main growth class of adult-sized gulliver trees (AG). A) Adult-sized gulliver type 1 (AG1) with extensive losses in crown biomass; note that a conventional stem-based allometric model would have missed the extensive canopy losses, while a purely canopy-based or remote sensing approach would underestimate the extensive stem's biomass; B) Adult-sized gulliver type 2 (AG2) which lost one out of its two big stems to disturbance topkill while the other stem remained rather undamaged and lives on; note that a remote sensing approach would probably not have linked the dead stem and its losses to the living stem; C) Adult-sized gulliver type 3 (AG3) which has lost its single main stem to topkill through elephant browsing and is now resprouting as a multi-stemmed shrub from the live root remains; only an individual-based method can explain the atypical shrub-like growth form in this tree species (*Burkea africana*); D) AG3 which has lost its main stem to topkill through fire and is now resprouting as a multi-stemmed shrub; only with an individual-based damage assessment can this very old gulliver individual be told apart from a younger sapling of similar canopy dimension and only then can biomass losses and regrowth potential be assessed reliably.

Fig. 2: In Fig. 2 we present photographic examples for the growth class of adult-sized gullivers trees and its sub-types (see details on growth classes below).

**Fig. 3:**
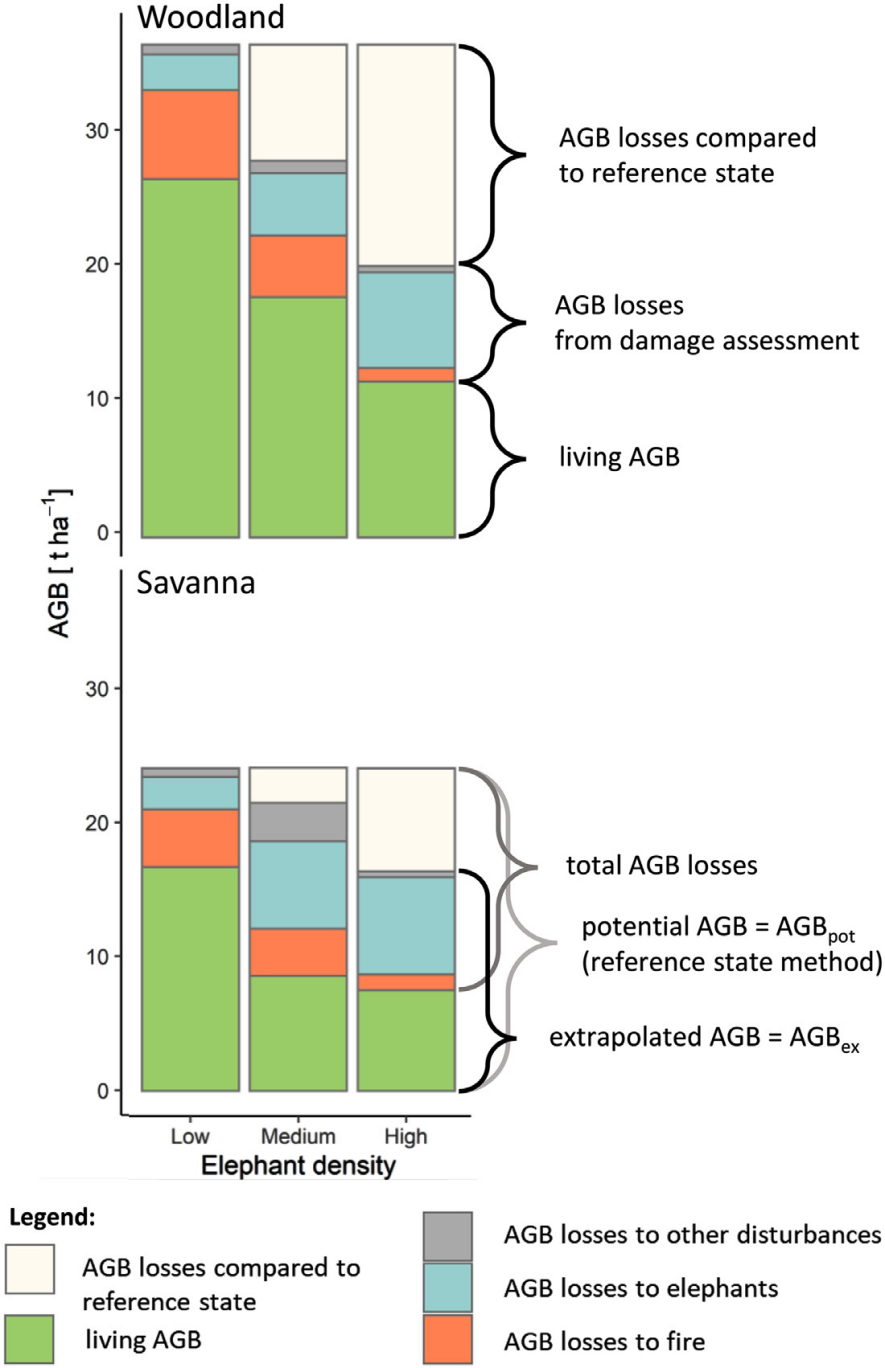
Mean aboveground biomass (AGB) and AGB losses to main disturbance agents per vegetation type and elephant density level. AGB_ex_ = assumed pre-disturbance AGB level as extrapolated from damage assessment, AGB_pot_ = maximum potential AGB level per vegetation type as derived from AGB_ex_ at the reference state of low elephant disturbance. ‘Other’ disturbances comprise woodcutting, storm, insect pests, and unidentifiable disturbance agents. Outlier plots were excluded here.

Fig. 3: In Fig. 3 we present a rough visualization of mean aboveground biomass (AGB) and AGB losses to main disturbance agents per vegetation type and elephant density level.

*Supporting Material*: This file contains supporting material illustrating the biomass estimation method with which the data in spreadsheet ‘data prop’ has been generated. All formulas are presented and detailed workflows of AGB estimation are illustrated for each growth class. A detailed illustration of workarounds for extrapolation of missing dendrometric proxies from measurable proxies is given as well.

## 2 Experimental Design, Materials and Methods

### 2.1 Study design

The data presented here is an exemplary dataset that relates to a research article [1], which presents a novel approach to estimate individual- and stand-level woody aboveground biomass (AGB) in disturbance-prone ecosystems such as drylands. The methodology consists of field sampling routines and workflows for a non-destructive estimation of AGB and AGB losses in woody vegetation, harnessing the archival function of trees for disturbances. The method was tested with the aid of large tree inventories collected along steep gradients of elephant disturbances in semi-arid savanna ecosystems located in Namibia's Zambezi Region. The dataset consists of the raw data on dendrometric proxies taken in the field, individual-level AGB estimates and AGB loss estimates generated with the proposed method, and species-wise mean values on specific woody density (SWD).

Our data were collected in savannas located in Namibia's Zambezi Region; for more information on the study area, see [1]. Sampling was stratified into two vegetation types (savanna and woodland) and three disturbance classes with low, medium, and high elephant densities; see Sandhage-Hofmann et al. (2021) [2] for details on this study design. Sites with ‘medium’ and ‘high’ elephant densities were located in the national parks Mudumu and Bwabwata, and least-disturbed sites with ‘low’ elephant densities were located in the respective adjacent communal conservancies Wuparo and Mashi. While the Mudumu-Wuparo set represented open, savanna-like vegetation (‘savanna’), the Bwabwata-Mashi set represented a more closed savanna woodland (‘woodland’), yielding a total of 6 sites (2 vegetation types x 3 elephant density levels each). In both national parks, areas of high elephant density were retrievable in close proximity (usually ≤ 1 km) to permanent water sources, while medium-density levels were found in greater distance [13,14]. Ten independent observation plots with a minimum distance of 80 m to each other and a size of 1000 m² (25 × 40 m) were sampled per site (6 sites x 10 plots = 60 in total). Sampling took place from September to November 2018, and from April to June 2019.

### 2.2 Data acquisition

#### 2.2.1 Sampling approach

The original study [1] presents and tests a sampling protocol for an assessment of woody individuals within a stand, including small and highly damaged ones. To this end, six growth classes were defined as the basis for class-specific measurement protocols i.e., three comparatively undamaged classes, and three heavily damaged ‘gulliver’ growth classes (see Fig. 1 for details and class definitions). While the original definition of a gulliver only refers to heavily damaged individuals which are seemingly juvenile and not reproductive [15,16], we extended this definition to encompass all heavily damaged individuals, irrespective of their true age or reproductive status. According to our definition, gullivers are heavily damaged woody individuals for which their height does neither reliably predict their age nor AGB (see Fig. 2 for examples).

As suggested by Kershaw et al. [17], a flexible sampling strategy with a nested plot design was used for inventories. Each of the 60 plots had a standard size of 1000m² on which all adult-sized, healthy trees were recorded. Nested within this plot (i.e. on a smaller subplot area, usually with a size of 750–1000 m²), all adult-sized gulliver trees were recorded. Again, nested within this first subplot, on a second subplot with an even smaller sampling area (usually within the range of 250-750 m²), all saplings were recorded in addition to the adult trees. Nested within that second subplot was the third, smallest subplot with fixed size of 100 m², on which we usually recorded every woody individual, including those belonging to juvenile growth classes.

The practical sampling procedure was as follows: After laying out the borders of the entire 1000 m² plot and of the smallest, fixed-sized subplot of 100 m², data recording started on this subplot. Here, all woody individuals were identified to species level, recorded with allometric measures, and subjected to a damage assessment. Only in cases where the abundance of juveniles was very high (> 100) the recording of juveniles was stopped after 40-60 m². On the remaining plot area, only saplings and adults were then recorded on the progressively larger subplots until a representative number (usually 15-20 individuals for all species) was reached. We recorded the respective subplot area for each growth class to allow subsequent upscaling from individual AGB values to a unit per area basis (Eq. (6)). This sampling design has been honed for dryland vegetation in particular because sparse woody vegetation with singular large trees requires large plot sizes [18] while very high numbers of small and juvenile individuals would make it extremely time-consuming to record all individuals on the full plot area [17]. Using a minimum height threshold for woody individuals to be recorded, as is often done in other ecosystems [12,19], was found to be impossible in our study, as with gullivers the height alone is an insufficient proxy for an individual's age due to severe and chronic disturbances in the ecosystem [14]. Using a minimum stem circumference for woody individuals to be recorded, as is often done in other ecosystems [19,20], was not possible in our study region as that would have excluded the extensive contribution of shrubs to overall AGB [1], [21].

Combining the growth class system with a nested plot design where the sampling area was flexibly decided for most growth classes allowed for an adjustment of the sampling effort according to plots’ population densities and species richness. The stratified sampling along growth classes required practical consideration on how large the sampling area for each class needs to be in each plot. Where species richness was low, and most trees and shrubs were damaged by the same disturbance agent in a similar manner smaller sampling areas were sufficient to represent the vegetation and the disturbance impacts therein. On the other hand, where species richness was high and woody growth had been affected by multiple disturbance agents larger sampling areas were required to record adequate data. For instance, plots with few species, an even distribution of individuals across growth classes, and a high number of juveniles was sufficiently represented by recording juveniles on a reduced sampling area of 50 m², further saplings and shrubs on 300 m², and highly damaged (‘gulliver’) adult trees on 500 m². In contrast, a plot with many species, a heterogenous growth class distribution, and a clumped and/or sparse vegetation was better represented by recording juveniles on a larger area (100–150 m²), saplings/shrubs on 500–750 m², and all adult trees – including highly damaged ones – on the full 1000 m² plot area. The guidelines of what was considered a ‘representative number of individuals’ [17] in each plot, has been determined along the following guiding questions:

• Is the part of the plot covered so far representative for the whole plot in regard to number and species composition of the growth class in question?
• Do we need to sample a bigger area to also reliably capture the damage levels and their heterogeneity for this growth class?
• How well does the sampled plot area represent its surrounding? Does it need to be enlarged to counterbalance patchy or clumped vegetation patterns or to cover a sufficient number of individuals?

When tree biomass estimations on an individual level are accumulated for a stand level, they are usually expressed on a unit per area basis e.g., as kg biomass ha^−1^ or t biomass ha^−1^ [19,22]. To upscale our individual-level biomass data, we needed to note the realized sampling area per growth class. For each growth class per plot, a factor for upscaling was then calculated by dividing 1 ha by the realized sampling area for each growth class and then multiplying individual biomass estimates by the respective upscaling factor; this yields values on a unit per area basis which were summed up per plot to express total AGB in the unit kg ha^−1^ or t ha^−1^.

Beyond the dataset presented here, our approach of a stratified sampling also allowed us to capture irregular sampling units like agricultural fields by measuring an entire field's area with a GPS device, recording all adult trees within the field and along the field margins, and combining that with suitable rectangular subplots for the sampling of juveniles and saplings.

#### 2.2.2 Allometric measurements

As the trees on our plots often had irregular growth forms which is typical for highly disturbed ecosystems [23], measuring circumference at breast height was not always possible. In these cases, we measured stem circumferences at various other heights to infer diameter at breast height (DBH) by own local calibrations [24]. In cases where stems were branching lower than 130 cm above ground level, the circumference was taken below the branching and the height at this alternative measure was recorded as well to calculate regressions between basal diameter, DBH, and height. For some heavily damaged trees, a second circumference measure was not derivable, hence a surrogate DBH (DBH_est_) was later estimated from their basal circumferences (see Supplementary Fig. 1). Also, if stem bases were partially missing, an educated guess had to be made of the basal circumference that was present before the severe damage occurred. In most cases, this was easy to achieve as the general curvature of the stem was still visible and a missing section was extrapolated.

Please note that, when adopting our methodology, the allometric measurement procedure could be further streamlined according to the targeted allometric models. Therefore, some of the measurements taken here might not be mandatory for other applications and allometric models, although taking full sets of measures for all individuals will render later application of future allometric models possible. It also allowed for us to have our own locally calibrated models of DBH ‒ height relationships, which is favorable [24].

We recorded separate sets of dendrometric proxies for the six growth classes, see Fig. 1 and Kindermann et al. [1]. For subadults, we recorded (i) height and (ii) the widest canopy diameter of living tissue and a second measure orthogonal to the first. For adults, we recorded (i) height of highest living tissue, and (ii) stem circumferences for all adult-sized stems at the base and at breast height (130 cm), if possible. These separate procedures for tree-like adults and shrub-like subadult individuals were necessary to enable the subsequent use of two allometric models. This was required because tree-like individuals (or part of individuals) are better captured by stem proxies while shrub-like individuals (or parts of individuals) are better captured by canopy dimension proxies [21].

For adult-sized gullivers, we also recorded the basal circumference of adult-sized dead stems (if any were present) and noted those stems as “dead”. Recording big dead stems may seem laborious but from our experience only required little extra effort while this information later became highly valuable to quantify lost biomass fractions (see below). For the special case that the living woody individual attached to such a dead stem contained no living adult-sized stems (growth class AG3; see Fig. 1 and Supplementary Fig. 2), we recorded canopy diameters instead, as was done in subadults. The reason is that the living biomass of the shrub-like parts of the individuals later needed to be estimated from the canopy dimensions, while the extensive biomass loss through topkill of the former tree-like growth could only be estimated from the dead stem's proxy measurements. This specialized estimation procedure was justified by the fact that a very high number of individuals belonged to this growth class [1].

#### 2.2.3 Damage assessment

For each woody individual in the tree inventory, a detailed damage assessment was conducted. The main disturbance agents in our study were recorded independently. These were fire damage, elephant browsing, browsing by other herbivores, woodcutting, and dieback due to abiotic stress. Other disturbance agents e.g., insect herbivory or storm damage, were subsumed under “other”, but if possible identified by a comment. Distinguishing the main disturbance agents was possible through their specific damage patterns. We used healthy trees and shrubs in comparison to damaged ones to estimate how much of the biomass had been lost in the latter. It was helpful to visualize what healthy individuals of the same species looked like (e.g., straight stem with a regular, well-proportioned crown) and then pay attention to the deviations. Scars of lost branches or firemarks on the bark were used as indicators for the causes that led to irregular growth forms of damaged trees. The damage assessment was performed by visually estimating the percentage of AGB lost to different disturbance agents [following 25]. A score of 0% damage was assigned to completely intact woody individuals without any apparent lost branches or scars. A damage estimate of 99% damage was assigned to individuals after total topkill. From our experience, the best way to ensure consistency in assessment was for the entire team to conduct joint assessments at the beginning of a field session, as practiced for other observer-dependent field records such as visual cover estimations [26]. We jointly estimated the percentage of biomass lost and calibrated our individual estimations against each other. Estimates from different researchers had only negligible variance after a short time of joint calibration.

Please note that adult-sized dead stems (> 15 cm basal circumference) attached to a living gulliver were not included into this estimation of percentage biomass lost. Instead, they were recorded independently, as their former biomass often exceeded the retained living biomass by orders of magnitude (see e.g. Fig. 2D). Dead adult-sized stems were measured with basal stem circumference and annotated as ‘dead’. The presumed topkill agent was identified, if necessary two joint topkill agents were listed. This procedure helped to stratify the disturbance losses on small living regrowth, which would only make up for < 1% if the dead adult-sized stem was to be counted in; by calculating lost biomass for the dead stem independently, it was possible to split up the total of 100% of living regrowth biomass to several disturbance agents. Please note that indicated biomass losses summed up over all disturbance agents cannot exceed 99%.

#### 2.2.4 Specific wood density

For AGB estimation with the allometric model of Chave et al. [12] we measured species’ specific wood density (SWD) following the ‘wood specific gravity’ protocol in Pérez-Harguindeguy et al. [3]. We sampled the stem wood of 2-20 individuals per species, using a 2-threaded increment borer (Haglöf Sweden®, inner diameter 5.15 mm). For sampling of wood cores, we selected healthy and rather straight stems, as otherwise the corer often became stuck. We aimed to sample both sapwood and core wood as they can have different specific weights in many tree species. Where drilling was not possible (i.e. in shrubs), we collected cylindrical stem sections instead, preferably a straight piece without any branching. In total, we took 412 samples from 65 species according to the species’ abundances. Bark was removed from the end of the core or peeled off the stem pieces, respectively. Each sample was measured in length, and five separate diameter readings along the sample were taken with calipers to determine wood fresh volume. Wood samples were stored in paper envelopes and later oven-dried (105 °C) until the weight was constant. Dry weight and fresh green volume were used to calculate SWD (dry weight per fresh volume), and measurements were averaged per species.

### 2.3 Estimation of aboveground biomass (AGB)

#### 2.3.1 Stocks vs losses

Unlike other studies on carbon storage, we not only aimed to quantify actual standing biomass stocks and carbon stored therein, but also took an interest in carbon losses to better understand joint processes of carbon storage and loss in a disturbed ecosystem. When adapting existing methods from standard protocols and creating our own workflows we realized, that quantifying stocks and estimating losses could not be covered by the same procedure, although these traits are interdependent. Part of our methodology therefore follows the rather straightforward sequence of measuring a living tree, estimating its biomass from an allometric model, and then deducting an estimated percentage of biomass lost to a recorded disturbance. For slightly damaged trees (< 30% biomass loss), this was the best procedure, but several problems arose when we applied the procedure to heavily damaged individuals, which we briefly report here with the respective methodological solutions.

*Problem 1.* A damaged tree's allometric measures did not fit the usual allometric relationships between height and stem measures [23]. For example, if severe elephant damages reduced a tree to half of its original height, its new height was relatively shorter compared to its stem diameter. As a solution, the stem diameter was used to inform us about the tree's original height, and the height of the standing stem allowed for an estimation of biomass loss.

*Problem 2.* While DBH is a widely used metric in forestry, carbon studies, and allometric models, it can be fraught with problems in disturbed ecosystems: stems were branching lower than the height of 130 cm or a stem was simply broken off, torn, or burnt below or at that height. Our solution here was to use basal stem circumference as the measurement threshold for the definition of adult trees. Moreover, whenever possible, we measured both the basal circumference and DBH for each stem with > 15 cm basal circumference. Additionally, from a healthy subset of trees in our dataset we derived a regression equation between basal circumference and DBH (Eq. (4)) and used this equation to determine DBH values (DBH_est_) for heavily damaged trees which were lacking a measurable DBH.

*Problem 3.* Damaged trees often displayed an atypical growth form when regrowing, and many species in our study region were observed to be growing in tree-like as well as shrub-like architectures [27]. Hence, an a-priori separation between shrub and tree species for an adoption of species-wise allometries was not possible. To address this problem, two different allometric models were applied across all species, with one for shrub-like individuals or plant parts, and another for tree-like individuals, respectively (see below). Hence, in some cases, the two models had to be combined to estimate an individual's biomass.

*Problem 4.* While small damages were deducted from the calculated biomass, huge damages were impossible to express in relation to living biomass, as they often exceeded the living biomass by orders of magnitude. As a solution, those damages were quantified separately, through circumference measures of dead stems which were fed into the allometric stem-model.

These problems and solutions illustrate that the calculation of standing biomass and the estimation of biomass losses to disturbances were two distinct procedures, yet not to be decoupled as the unit of observation still was a woody individual.

#### 2.3.2 Estimation procedure of AGB and AGB losses

An estimation procedure for AGB and AGB losses was designed for each of the six growth classes; see Supporting Material (Supplementary Table 2 and Supplementary Fig. 2) for further details. For the comparatively undamaged non-gulliver growth classes, AGB estimation was done with the aid of a stem-based model for adults, and with a canopy-based model for subadults. Please note that the two specific models used here may be replaced by whatever local stem- and canopy-based models you prefer or will be available in the future. Separate estimation workflows were performed for four subtypes of adult-sized gullivers (AG) to account for their distinct, irregular growth forms shaped by severe disturbances (see Fig. 2 and Supplementary Fig. 2). The first type (AG1) had more substantial biomass losses than non-gulliver adults (≥ 30%), but still a regular growth. Here we only added a height correction to the standard method of AGB calculation to reflect pre-disturbance height. The second type (AG2a) had both dead and living stems. As living stems bore comparatively undamaged crowns, a height correction was not necessary for them. Here, biomass losses in the form of dead stems were estimated based on their basal stem diameter. From a dead stem we were able to measure at least one proxy (basal circumference) which still held information about the tree's pre-damage dimensions. Adult-sized gullivers of type AG2b showed a combination of damages seen in the former two subtypes i.e., they displayed both severe damages in living stems’ canopy and had dead stems. We thus performed a height correction for living canopies (as done for AG1) and then estimated dead stems’ biomass (as done for AG2a). Finally, AG3 gullivers had a shrub-like appearance after having experienced topkill of their former main stem and had at least one major dead stem. The living biomass was thus a regrowth from the root system. In this case, AGB losses were estimated as for dead stems in types AG2a and AG2b, while living AGB of the shrub-like part was estimated via the canopy-based allometric model also used for AGB estimation in subadults. For the specific formulas applied in the procedure see the next section. A detailed visual workflow of AGB and AGB loss estimation for each growth class is provided in the Supporting Material.

#### 2.3.3 Formulas

For AGB estimation of shrubs and subadult growth classes, we used the generic, canopy-based model developed by Conti et al. (2019) [21] (Eq. (1)). It estimates aboveground biomass (AGB, in [kg]) from mean crown diameter (CD, in [m]) and height (h, in [cm]). The canopy-based model was also used to estimate living AGB fractions of adult-sized gullivers, where these fractions met the size criteria of subadults (AG3).

(1) AGB=exp(−0.370+1.903Ln(CD)+0.652Ln(h))*1.403

For adult trees, we used the generic, stem-based model by Chave et al. (2014) [12]. It estimates aboveground biomass (AGB, in [kg]) from specific wood density (SWD, in [g cm^−3^]), stem diameter at breast height (DBH, in [cm] at 1.3 m above ground level) and the tree height (h, in [m]), see (Eq. (2), [12]).

(2) $$\text{AGB}=0.0673*{\left(\text{SWD}*\text{DB}H^{2}*h\right)}^{0.976}$$

For adult-sized gulliver trees (AG) we first reconstructed their pre-disturbance height (h_est_, in [cm]) from their DBH by using a regression developed from adult non-gulliver trees (AA) in our dataset (Eq. (3), see [1]), before feeding these values into the stem-based model. In cases where h_est_ < h, we kept the initial height reading h.

(3) $$h_{\mathrm{est}}=\exp\left(4.72595+0.63385*\mathrm{LN}\left(\mathrm{DBH}\right)\right)\;\;\left(R^{2}=0.75\right)$$

For damaged and dead stems where a DBH reading was not possible, we took a basal circumference (in [cm]) and estimated a pre-disturbance DBH (DBH_est_, in [cm]) by a regression, again built from adult non-gulliver trees (AA) in our own dataset (Eq. (4), see [1]):

(4) $$\text{DB}H_{\text{est}}=0.7968*\frac{\text{basal}\;\text{circumference}}{\pi }\;\;\;\left(R^{2}=0.9639\right)$$

For the few cases of stems in which neither DBH nor basal circumference were applicable (e.g. a tree that was branching below or directly at 130 cm and also had been damaged heavily from fire at the base), we took a circumference reading at an alternative height (circ(hx), in [cm]) along the stem, and the height where this reading was taken (hx, in [cm]). From these measurements we first extrapolated a basal circumference (see Eq. (5), which was derived from our own data):

(5) $$\text{basal}\;\text{circumference}=\frac{130\;*\;circ\left(hx\right)\;}{130-0.2032\;*\;hx}$$

From this reconstructed basal circumference, DBH_est_ was again estimated through Eq. (4). This DBH_est_ was also used to extrapolate a former height h_est_ (using Eq. (3)) where necessary (i.e. for living stems in AG1 and AG2b, or dead stems), and both proxies were used to calculate AGB according to Eq. (2). We advise to newly establish the latter three formulas (Eqs. (3)–(5)) whenever our estimation procedure is adapted to other regions and species communities. They can easily be generated from existing measurements in rather undisturbed trees of the same data collection. Please note, that in our example Eq. (3) is a power law function and Eq. (4) is a linear regression and their fit was quite convincing (see [1] and Supplementary Fig. 2 therein), but depending on species composition other regression types might deliver a better fit.

For trees with multiple adult-sized stems, we calculated individual stems’ AGB with the same height, but with their respective measured or estimated DBH. Multiple stems’ AGB was then summed up per individual. As we had used different sampling areas per growth class, we used an upscaling factor (Eq. (6)) to express individual AGB on a per unit area basis (in kg ha^−1^); see Kershaw et al. [17] and more detailed explanations above in section ‘Sampling approach’. The plot-wise sum of these individually upscaled values then reflects stand-level AGB on a unit per area basis.

(6) $$\text{upscaling}\;\text{factor}=\;\frac{1\;\text{ha}}{\text{area}\;\text{covered}\;\text{for}\;\text{respective}\;\text{growth}\;\text{class}}$$

To calculate AGB losses for living stems and in crowns, we multiplied AGB with the estimated proportion of biomass lost due to different disturbance agents. As canopy-based models and stem-based models differ with respect to the way how they incorporate AGB losses (see [1]), AGB estimated from the canopy-based model already reflects the actual damaged state, whereas with stem-based models crown AGB losses have to be subtracted first to obtain actual standing AGB.

In dead stems, former AGB was quantified via the generic model by Chave et al. [12] with the workaround of first estimating an unmeasurable DBH from basal circumference (see Eq. (4)). A dead stem's former AGB was then added to the AGB losses that were visually estimated for the respective disturbance that had also caused the topkill. Details on calculation procedures are presented in the Supporting Material. Where AGB losses are displayed on a unit per area basis they have been multiplied by the upscaling factor, as was done for AGB.

#### 2.3.4 Task-wise overview of our methodology

To summarize the detailed information on methods and procedures above, this is a list of the nine tasks that were performed to generate the dataset presented in this paper. Tasks 1-3 are fieldwork tasks, while the remaining six tasks are lab and office tasks.

 Task 1 – determine the appropriate size of subplots (nested within 1000 m² plots) for the sampling of growth classes:
  • As a default, consider a sampling area of 100 m² for the sampling of juveniles (corresponding to the fixed-size subplot), while saplings and damaged adults are recorded on larger subplots that represent a known fraction of the plot, and non-gulliver adults are by default recorded on the whole 1000 m² plot
  • If woody individuals are very abundant, but uniformly disturbed, reduce the sizes of nested subplots for the respective growth classes
  • Where individuals are sparse and/or disturbance patterns are unevenly distributed, increase the size of the respective subplot until recorded woody individuals reflect disturbance patterns and demography of the surrounding landscape
  • Make sure to sample a sufficient number of individuals per species; we suggest choosing sampling areas so that a minimum 15-20 individuals are sampled per species, unless species are rare, and individuals are solitary
 Task 2 – record woody individuals of different growth classes on progressively larger subplots:
  • Start with the 100 m² subplot (see above) where usually all individuals of all sizes and age classes are recorded: record their species name, growth class and dendrometric proxies, and estimate the percentage of biomass lost to each disturbance agent
  • Progress with inventories and damage assessments to the next larger subplot (with its size flexibly decided), where you stop to record juvenile individuals but continue to record all other growth classes
  • In case you chose different sampling areas for sapling and adult growth classes, create another cut-off and only record adult growth on the extended area; note final sampling areas which were realized within each plot for each growth class
 Task 3 – take wood samples for woody species on the plot to estimate specific wood density (SWD):
  • Check for all woody species that are present on the plot if wood samples were already taken for ten individuals; if yes, wood sampling can be skipped for this species
  • For species sampled with < 10 individuals in total, select a healthy individual, and extract two wood cores from the stem(s) or cut 2–3 stem pieces
 Task 4 – process wood samples to estimate SWD:
  • Oven-dry wood samples and take their dry weight to determine SWD following Pérez-Harguindeguy et al. (2013) [3]
  • Fill data gaps with SWD values from existing databases
 Task 5 – digitize data into spreadsheets and analyze a subset of healthy individuals:
  • Create a subset of all non-gulliver adult trees in your total dataset
  • Perform data exploration techniques following Zuur et al. [28]
  • Use this subset to parameterize Eqs. (3)–(5) for your dataset
 Task 6 – fill in missing allometric size proxies:
  • For badly damaged stems, a missing DBH value needs to be deduced from actually measured basal stem circumference via Eq. (4)
  • The same procedure needs to be performed for dead stems, which were only measured at the base
  • For adult gullivers, a pre-disturbance height needs to be estimated from the stem proxies via Eq. (3)
 Task 7 – estimate AGB fractions according to the workflow designed for its respective growth class (see Supporting Material, Supplementary Fig. 2, and Supplementary Table 2):
  • For tree-like individuals, estimate pre-disturbance AGB first and then deduct recorded AGB losses to gain post-disturbance AGB (i.e., actually standing, live AGB)
  • For shrub-like individuals, the initial AGB estimation yields the post-disturbance value from which a pre-disturbance value can be estimated through reverse damage assessment (see Supporting Material, Supplementary Fig. 2)
  • Estimate pre-topkill AGB of recorded dead stems
 Task 8 – calculate AGB losses per disturbance agent:
  • Fractionate estimated AGB loss from canopies according to percentage losses recorded visually in the field: multiply estimated pre-disturbance AGB by percentage loss recorded for that disturbance agent to gain AGB lost to that disturbance agent specifically
  • Use the disturbance agent responsible for topkill in dead stems to allocate this additional AGB loss to a disturbance agent
  • Aggregate AGB losses created by different disturbance agents into ‘total AGB losses’
 Task 9 – upscale individual values to unit per area:
  • Multiply each individual's living AGB by the upscaling factor (Eq. (6)) needed for the respective plot and growth class
  • Also use scale-up factors on all other AGB partitions e.g., AGB losses and AGB of dead stems
  • Sum up these values per plot to gain overall AGB and AGB losses per plot on a unit per area basis
  • For individuals growing on the plot edge, correct for the tree's or shrub's fraction falling inside the plot

Following the procedures listed above we were able to quantify woody AGB in a naturally disturbed ecosystem and also attribute fractions of AGB lost to specific disturbance agents like elephant browsing or wildfire (see Fig. 3). Results indicate that AGB losses to wildfire are decreasing with increasing elephant densities (Fig. 3). This trend was observed for both savanna and woodland vegetation, although baseline woody AGB levels of both vegetation types were found to be markedly different (Fig. 3). We hope to have demonstrate the added value of integrating the two procedures of biomass quantification and disturbance-specific biomass loss estimation for woody biomass in savanna and dryland ecosystems.

## CRediT authorship contribution statement

**Liana Kindermann:** Conceptualization, Visualization, Methodology, Data curation, Software, Formal analysis, Writing – original draft, Writing – review & editing. **Magnus Dobler:** Data curation, Software, Formal analysis, Writing – original draft, Writing – review & editing. **Daniela Niedeggen:** Data curation, Software, Formal analysis, Writing – original draft, Writing – review & editing. **Ezequiel Chimbioputo Fabiano:** Visualization, Resources, Writing – original draft, Writing – review & editing. **Anja Linstädter:** Conceptualization, Visualization, Methodology, Data curation, Writing – original draft, Writing – review & editing.

## Declaration of Competing Interest

The authors declare that they have no known competing financial interests or personal relationships which have or could be perceived to have influenced the work reported in this article.

## Data Availability

Dataset on Woody Aboveground Biomass, Disturbance Losses, and Wood Density from an African Savanna Ecosystem (Original data) (Mendeley Data).

## Appendix Supplementary materials

Image, application 1
