# Supplementary material for: Dataset on woody aboveground biomass, disturbance losses, and wood density from an African savanna ecosystem
Source: Data Brief. 2022 Apr 11;42:108155. doi: 10.1016/j.dib.2022.108155 (PMC9062271; doi:10.1016/j.dib.2022.108155)
Supplement: Supplementary file 1 [file mmc1.docx]

Supporting Material

To Data in Brief article:
Dataset on Woody Aboveground Biomass, Disturbance Losses, and Wood Density

Authors: Liana Kindermann, Magnus Dobler, Daniela Niedeggen, Ezequiel Chimbioputo Fabiano, Anja Linstädter

*Supplementary Table 1. Biomass partitions which a tree or shrub can be broken down to; these were independently calculated and then combined according to growth class specifications to reflect biomass stocks and losses. AGB = aboveground biomass, act = actually alive, calc = calculated by stem measures, reclost = recorded losses from damage assessment, dead = dead adult-size stems, ex = extrapolated from alternative allometric measures. Table taken from [1].*

| **Acronym** | **Biomass Partition** | **Description** |
| --- | --- | --- |
| **AGB_act_** | Actual AGB | Trees: What remains of AGB_calc_ after crown damages are deducted |
|  |  | Shrubs and shrub-like gulliver regrowth: biomass as represented by their (damaged) canopy measurements; directly estimated with formula by Conti et al. (2019) [2] |
| **AGB_reclost_** | AGB losses recorded during damage assessment | Trees: Estimated biomass losses (crown, branches) in relation to AGB_calc_; deducted % damages from AGB_calc_ (as AGB_calc_ was calculated from undamaged stem measures and (corrected) height) |
|  |  | Shrubs: Estimated biomass losses (branches) in relation AGB_act_; is added to AGB_act_ to obtain pre-disturbance AGB (AGB_ex_) as AGB_act_ was calculated from damaged crown dimensions |
| **AGB_dead_** | AGB of dead stems (on a living individual) | Dead stems attached to a living gulliver; this biomass is calculated independently from AGB_calc_ by dead stems’ own stem measures |
| **AGB_calc_** | AGB as calculated from allometric measures by Chave et al. (2014) [3] formula | Adult-sized trees only; biomass as represented by their (undamaged) allometric measures (height in AGs was corrected to h_est_); not incorporating the visible losses in e.g. crown biomass;  → close to what usual studies would have done, but more accurate than that, as we also included damaged stems which had no proper DBH (by estimating DBH_est_ from their basal circumference, see Supplementary Figure 1) |
| **AGB_ex_** | Extrapolated pre‑disturbance AGB | Hypothetical AGB of for an individual if it were undamaged: - for undamaged and slightly damaged adults (AA): AGB_ex_ = AGB_calc_  - damaged adults with a dead and a living stem (AG2a and AG2b): AGB_ex_ = AGB_calc_ + AGB_dead_  - for sub-adult classes (JJ, JG, SS, SG): AGB_ex_ = AGB_act_ + AGB_reclost_  - for shrub-like gullivers with a dead stem (AG3):   AGB_ex_ = AGB_act_ + AGB_reclost_ + AGB_dead_ |

*Supplementary Table 2. Formulas and biomass partitions combined to calculate each growth class’ AGB and AGB losses; auxiliary color coding according to Supplementary Figure 1 and Supplementary Figure 2 below; AGB = aboveground biomass, act = actually alive, calc = calculated by stem measures, reclost = recorded losses from damage assessment, dead = dead adult-size stems, est = estimated from alternative allometric measures. Table taken from [1].*

| **Growth class** | **AGB_calc_** | **AGB_dead_** | **AGB_reclost_** | **AGB_act_** |
| --- | --- | --- | --- | --- |
| **JJ, SS, JG, SG** | **----** | **----** | **AGB_act_ * biomass loss %** | **Conti et al. (2019) [2] eq. based on CD and h** |
| **AA** | **Chave eq. based on DBH, h  & SWD** | **----** | **AGB_calc_ * biomass loss %** | **AGB_calc_ - AGB_reclost_** |
| **AG1** | **Chave eq. based on DBH, h_est_ & SWD** | **----** | **AGB_calc_ * biomass loss %** | **AGB_calc_ - AGB_reclost_** |
| **AG2a** | **Chave eq. based on DBH, h  & SWD** | **Chave (2014) eq.; based on DBH_est_, h_est_ and SWD** | **AGB_calc_ * biomass loss % + AGB_dead_** | **AGB_calc_ – (AGB_calc_ * biomass loss %)** |
| **AG2b** | **Chave eq. based on DBH, h_est_ & SWD** | **Chave (2014) eq.; based on DBH_est_, h_est_ and SWD** | **AGB_calc_ * biomass loss % + AGB_dead_** | **AGB_calc_ – (AGB_calc_ * biomass loss %)** |
| **AG3** | **----** | **Chave (2014) eq.; based on DBH_est_, h_est_ and SWD** | **AGB_act_ * biomass loss % + AGB_dead_** | **Conti et al. (2019) [2] eq. based on CD and h** |


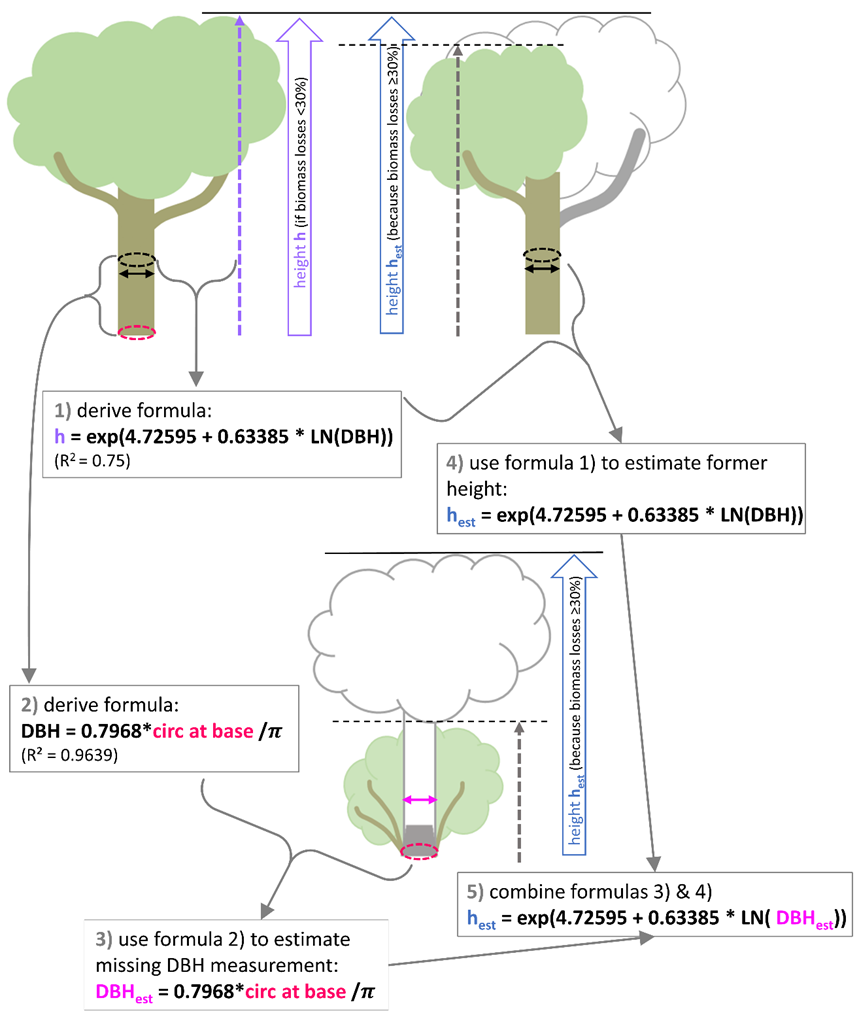


***Supplementary Figure 1:*** *Measuring and inferring size parameters in adult trees of different damage conditions. While undamaged and slightly damaged Adults (AA) are measured with their real height and DBH, adult-sized Gullivers (AG) require workarounds, depending on how severely the damages altered their crown and stem properties: From AAs in the dataset 1) a DBH-height relationship, and 2) a relation between basal stem circumference and DBH were inferred. These formulas were used to calculate missing height and stem dimensions: For AGs which had been damaged so severely that a major stem was lost, 3) a former DBH could be estimated (DBH_est_) from the basal circumference of the stump; 4) original pre-disturbance height of AGs can be estimated from actually measured, but also inferred, DBH values. Through this procedure, misleading height measurements (grey broken arrows) could be discarded and instead lost biomass was calculated by making use of the former height information contained in gulliver trees’ basal circumferences. Broken lines and arrows symbolize field measurements; solid lines and arrows symbolize values used for AGB estimations; AGB = aboveground biomass, DBH = stem diameter at breast height; est = estimated, h = height; AGB in [kg], DBH and h in [cm]. Figure taken from [1].*

***Supplementary Figure 2:*** *Detailed visual workflow of AGB and AGB loss estimation per growth class. Growth classes are abbreviated as: AA = undamaged and slightly damaged adult trees, AG = adult-sized gulliver trees, JJ = undamaged and slightly damaged juveniles; JG = juvenile-sized gullivers, SS = undamaged and slightely damaged saplings and shrubs, SG =  sapling and shrub gullivers; AGB = aboveground biomass, act = actually alive, calc = calculated by stem measures, CD = mean canopy diameter, dead = dead adult-size stems, DBH = diameter at breast height, est = estimated from alternative allometric measures, h = height, reclost = recorded losses from damage assessment.* *Figure taken from [1].*

| **JJ, JG, SS, SG** |  |
| --- | --- |
| **Icons** | 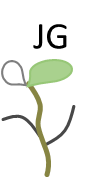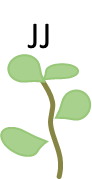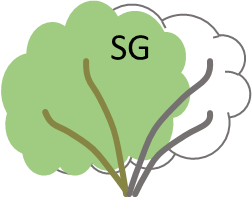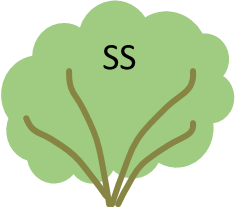 |
| **Size parameters** | 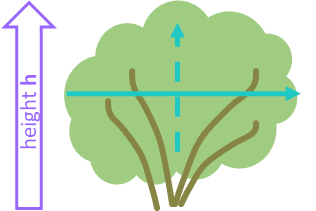Mean Canopy Diameter (**CD**) and height (**h**) |
| **Biomass partitions** | 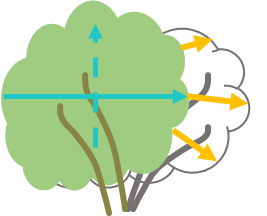**AGB_reclost_ = AGB_act_* recorded losses (%)** |
|  | **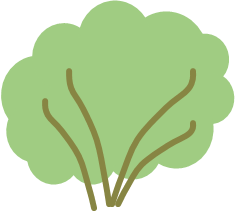AGB_act_** **= exp(−0.370 + 1.903 Ln(CD) + 0.652 Ln(h))*1.403**  (formula by Conti et al. (2019)) |

*Supplementary Figure 2 continued.*

| **AA** | undamaged | slightly damaged |
| --- | --- | --- |
| **Icon** | 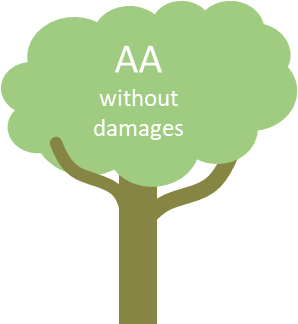 | 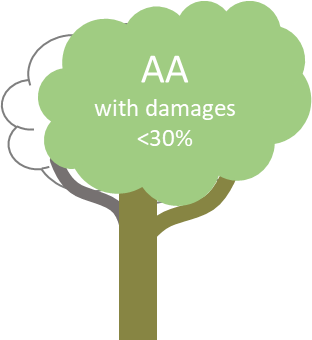 |
| **Size parameters** | Height (h) and DBH as measured in the field  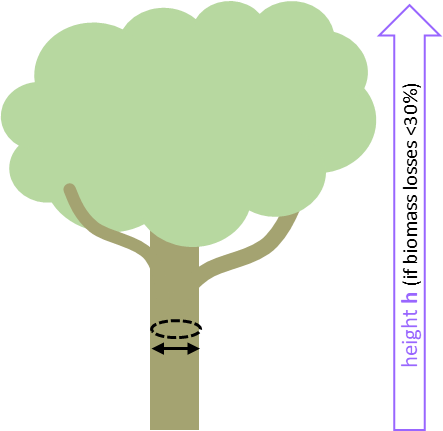 | |
| **Biomass partitions** | Estimating AGB as represented by stem properties and height:  **AGB_calc_** **= 0.0673 * (SWD * DBH^2^ * h)^0.976^**  (formula by Chave et al. (2014)) | |
|  |  | Calculating AGB lost to damages according to damage assessment:  **AGB_reclost_ = AGB_calc_ * recorded losses (%)**  **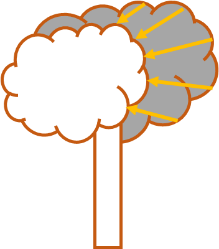** |
|  | Actually living biomass for undamaged individuals:  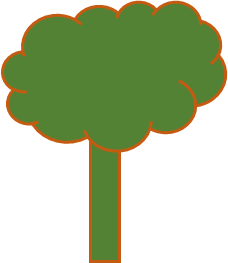**AGB_act_ = AGB_calc_** | Actually living biomass for slightly damaged individuals:  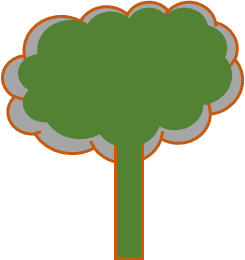 **AGB_act_ = AGB_calc_ - AGB_reclost_** |

*Supplementary Figure 2 continued.*

| **AG1** | Heavily damaged, but in crown only |
| --- | --- |
| **Icon** | 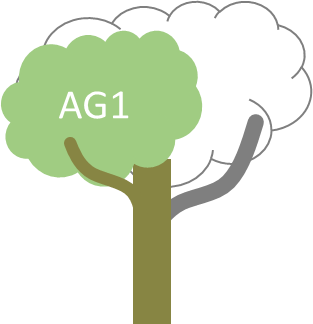 |
| **Size parameters** | 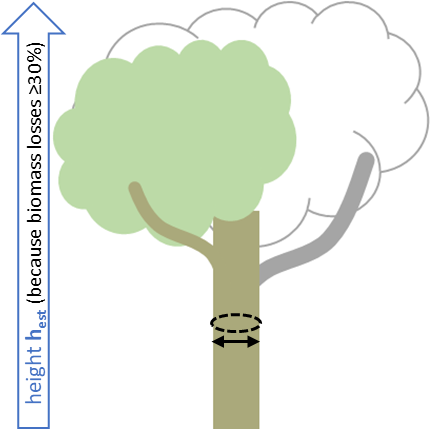DBH as measured in the field; former height (**h_est_**) estimated from DBH |
| **Biomass partitions** | Estimating AGB as represented by stem properties and corrected height:  **AGB_calc_** **= 0.0673 * (SWD * DBH^2^ * h_est_)^0.976^**  (formula adopted from Chave et al. (2014)) |
|  | **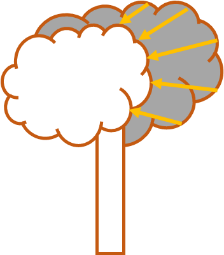**Calculating AGB lost to damages according to damage assessment:  **AGB_reclost_ = AGB_calc_ * recorded losses (%)** |
|  | Actually living biomass retained after damages:  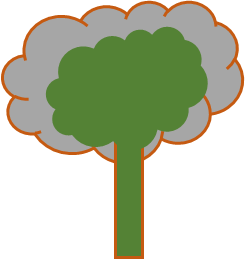 **AGB_act_ = AGB_calc_ - AGB_reclost_** |

*Supplementary Figure 2 continued.*

| **AG2a** | Heavily damaged, but all damage in form of a dead stem | |
| --- | --- | --- |
| **Icon** | 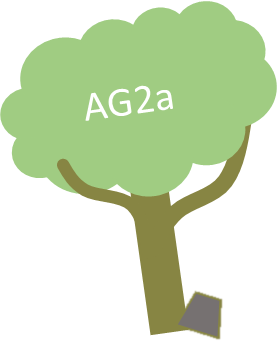 | |
| **Size parameters** | 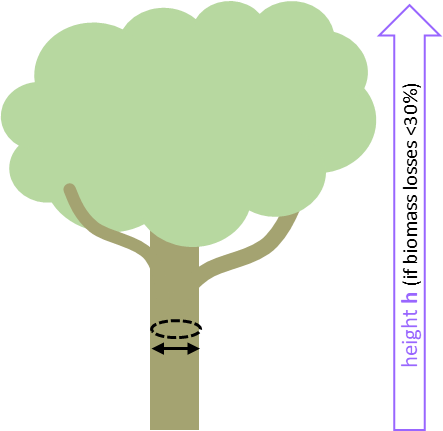Height (**h**) and DBH as measured in the field for the living stem | 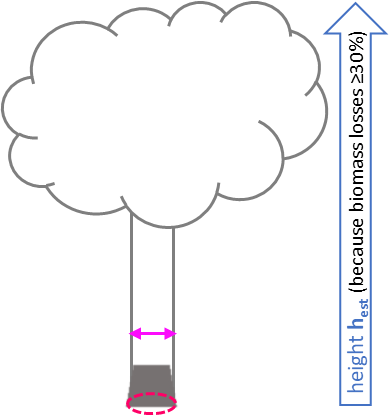Former DBH (**DBH_est_**) as estimated from dead stem’s basal circumference; former height (**h_est_**) estimated from **DBH_est_** |
| **Biomass partitions** | Estimating AGB of the living stem as in AAs:  **AGB_calc_** **=**  **0.0673 * (SWD * DBH^2^ * h)^0.976^**  (formula of Chave et al. (2014)) | Estimating AGB lost within the dead stem from estimated dendrometrics:  **AGB_dead_ =**  **0.0673 * (SWD * DBH_est_^2^ * h_est_)^0.976^**  (formula adopted from Chave et al. (2014) |
|  | _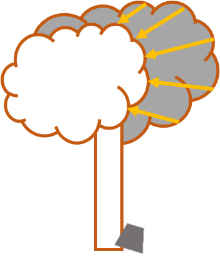_Calculating damages on the living stem:  **AGB_reclost_ = AGB_calc_ * recorded losses (%) + AGB_dead_** | |
|  | Without damges on the live stem:  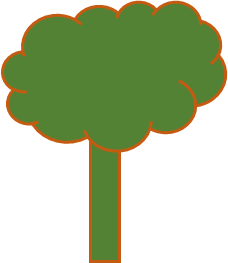 **AGB_act_ = AGB_calc_** | With minor damages on the live stem:  **AGB_act_ = AGB_calc_ - AGB_reclost_**  **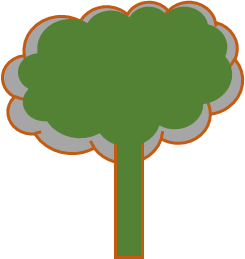** |

| **AG2b** |  | |
| --- | --- | --- |
| **Icon** | 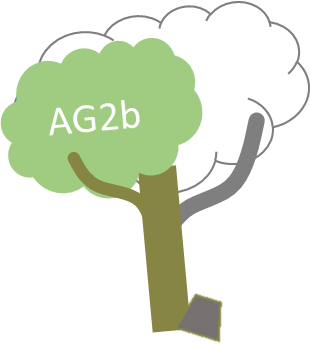 | |
| **Size parameters** | DBH as measured in the field; former height (**h_est_**) estimated from DBH for the living stem.  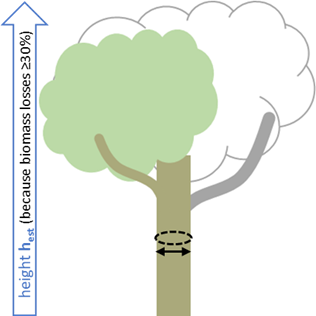 | 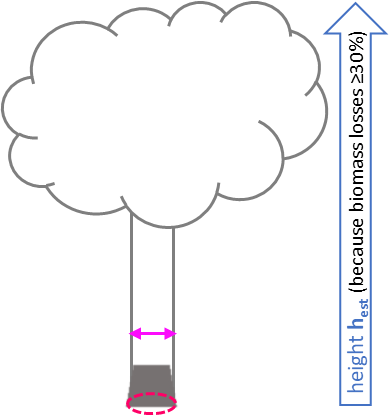Former DBH (**DBH_est_**) as estimated from dead stem’s basal circumference; former height (**h_est_**) estimated from **DBH_est_**. |
| **Biomass partitions** | **AGB_calc_** **=**  **0.0673 * (SWD * DBH^2^ * h_est_)^0.976^**  (formula of Chave et al. (2014)) | **AGB_dead_ =**  **0.0673 * (SWD * DBH_est_^2^ * h_est_)^0.976^**  (formula adopted from Chave et al. (2014)) |
|  | **_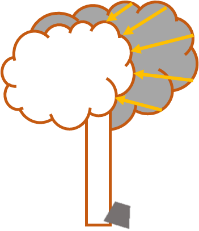_**  **AGB_reclost_ = AGB_calc_ * recorded losses (%) + AGB_dead_** | |
|  | 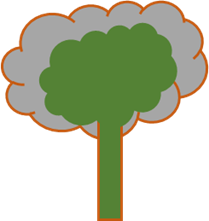 **AGB_act_ = AGB_calc_ - AGB_reclost_** | |

*Supplementary Figure 2 continued.*

| **AG3** |  | |
| --- | --- | --- |
| **Icon** | 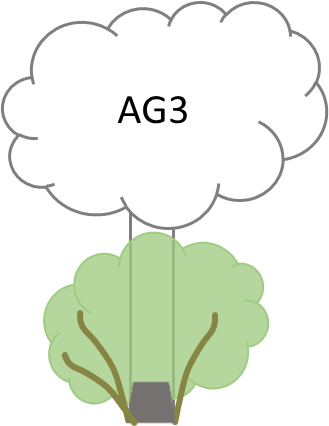 | |
| **Size parameters** | Mean Canopy Diameter (**CD**) and height (**h**) for the living shrub-like part of the gulliver tree.  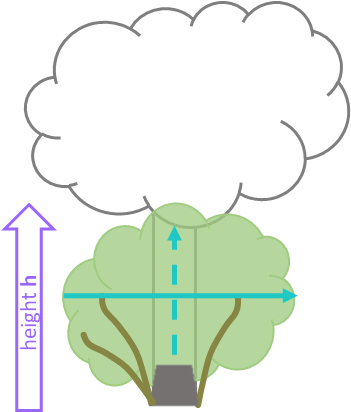 | Former DBH (**DBH_est_**) as estimated from dead stem’s basal circumference; former height (**h_est_**) estimated from **DBH_est_** for calculating dead stem’s biomass.  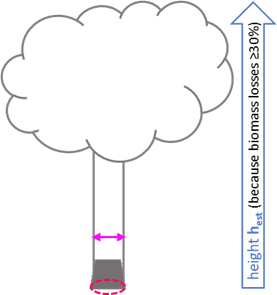 |
| **Biomass partitions** | **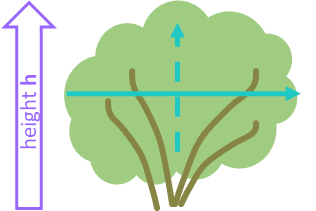AGB_act_** **= exp(−0.370 + 1.903 Ln(CD) + 0.652 Ln(h))*1.403**  (formula by Conti et al. (2019)) | **AGB_dead_ =**  **0.0673 * (SWD * DBH_est_^2^ * h_est_)^0.976^**  (formula adopted from Chave et al. (2014)) |
|  | **AGB_reclost_ = AGB_act_* recorded losses (%) + AGB_dead_** | |

*Supplementary Figure 2 continued.*

**References**

[1] L. Kindermann, M. Dobler, D. Niedeggen, A. Linstädter, 2022. A new protocol for estimation of woody aboveground biomass in disturbance-prone ecosystems. Ecol. Indic. 135, 108466.

[2] G. Conti, L.D. Gorné, S.R. Zeballos, M.L. Lipoma, G. Gatica, E. Kowaljow, J.I. Whitworth-Hulse, A. Cuchietti, M. Poca, S. Pestoni, P.M. Fernandes, 2019. Developing allometric models to predict the individual aboveground biomass of shrubs worldwide. Glob. Ecol. Biogeogr. 28, 961-975.

[3] J. Chave, M. Réjou-Méchain, A. Búrquez, E. Chidumayo, M.S. Colgan, W.B.C. Delitti, A. Duque, T. Eid, P.M. Fearnside, R.C. Goodman, M. Henry, A. Martínez-Yrízar, W.A. Mugasha, H.C. Muller-Landau, M. Mencuccini, B.W. Nelson, A. Ngomanda, E.M. Nogueira, E. Ortiz-Malavassi, R. Pélissier, P. Ploton, C.M. Ryan, J.G. Saldarriaga, G. Vieilledent, 2014. Improved allometric models to estimate the aboveground biomass of tropical trees. Global Change Biol. 20, 3177-3190.
